# Foreign Bodies in Lower Airway in Children: Brief Review and Clinical Experience

**DOI:** 10.3390/children12010067

**Published:** 2025-01-07

**Authors:** Stoyan Markov, Petya Markova, Ivanka Karavelikova, Hristina Halacheva

**Affiliations:** 1 Department of Otorhinolaryngology, Medical University of Plovdiv, 4002 Plovdiv, Bulgaria; 2 Department of Otorhinolaryngology, University Hospital “St. George“ Plovdiv, 4000 Plovdiv, Bulgaria; hristina.halacheva.16@trakia-uni.bg; 3 Department of Pediatrics, Medical University of Plovdiv, 4002 Plovdiv, Bulgaria; petya.markova@mu-plovdiv.bg (P.M.); ivanka.karavelikova@mu-plovdiv.bg (I.K.); 4 Department of Pediatrics, University Hospital “St. George”, 4000 Plovdiv, Bulgaria

**Keywords:** foreign body aspiration, airway foreign body extraction, pediatric bronchoscopy

## Abstract

Background: Foreign body aspiration is a preventable occurrence that carries a high risk of mortality in the pediatric population. Clinically, foreign body aspiration manifests as cough, followed by choking, which might not be given any consideration by the caregivers of the child. An episode of sudden wheezing can also raise the suspicion of a foreign body in the lower respiratory tract. The clinical findings depend on the type, size, and localization of the foreign body and include persistent cough, localized airway resistance, localized or diffuse wheezing, and difficulty breathing. A bronchoscopy is the procedure of choice for the removal of foreign bodies. Flexible bronchoscopy is increasingly being used as the initial diagnostic procedure in children with an uncertain history of choking, in the absence of physical and radiological lung changes, and in chronic complaints requiring the exclusion of a foreign body in the airways. Thus, the aim of this study was to describe our clinical experience with lower respiratory tract foreign body extraction in children over a period of five years. Materials and Methods: Over a 5-year period, 154 patients under the age of 18 underwent a bronchoscopy due to a suspected foreign body in the lower respiratory tract. Of these patients, 92% had an incident leading to acute respiratory distress, and 8% had no definite data on such an event in the anamnesis. Results: A foreign body in the respiratory tract was found in and extracted from 50 patients, and foreign bodies were absent in 104 of the cases. Conclusions: If a foreign body enters the lower respiratory tract, immediate and adequate actions are required to solve the problem. A bronchoscopy should be conducted in every suspected case of foreign body aspiration.

## 1. Introduction

The aspiration of foreign bodies and their entry into the lower respiratory tract is an emergency, especially in childhood, and in some cases, leads to acute respiratory distress and sudden death. The incidence of aspiration is not declining [1]. The mortality associated with this pathology is still high despite the progress in the emergency procedures related to foreign body aspiration [1,2].

More than three-quarters of foreign body aspiration cases occur in children under 3 years of age [3,4] due to the following reasons:

1. Failure of larynx closure through the laryngeal reflex.
2. Habits such as placing objects in the mouth, or simultaneously feeding and playing.
3. Immature swallowing reflex.
4. Parental lack of information regarding certain objects that can be aspirated, such as small toys and certain types of food, with predisposing factors [1].

### Signs, Symptoms, Diagnosis, and Treatment of Aspirated Foreign Body

Severe coughing, wheezing, dyspnea, or stridor are the main symptoms of a foreign body entering the lower respiratory tract [5]. This acute episode may escape adults’ attention as symptoms usually disappear or largely subside the moment the foreign body stops moving. That is how the cause of subsequent problems may remain unknown for a long time.

Small foreign bodies that remain motionless after entering the lower respiratory tract may produce no objective symptoms for days, and sometimes weeks. The development of pneumonia is usually the first sign of the presence of a foreign body, and its recurrence is an alarming sign.

Symptoms do not disappear when large foreign bodies enter the lungs and obstruct a large bronchus (main or lobar); breathing is weakened or absent in the area behind the foreign body. Auscultation reveals emphysematous (valvular mechanism of obstruction) or atelectasis (complete obstruction of the corresponding bronchus) changes. A balloting foreign body in the trachea results in the characteristic auscultatory sound known as the “champagne cork” symptom, which appears due to the impact of the foreign body on the vocal cords and carina of the trachea. Auscultatory changes may not be detected, especially in the first hours or days after aspiration, if the air freely passes the foreign body.

The exact type of foreign body, biological or not, that has entered the respiratory system must be determined because those of biological origin swell. This swelling leads to the gradual transformation of an incomplete bronchial obstruction into a total bronchial obstruction, representing the pathophysiological mechanism through which the progression to respiratory failure occurs due to the aspiration of a foreign body.

**Figure 1:**
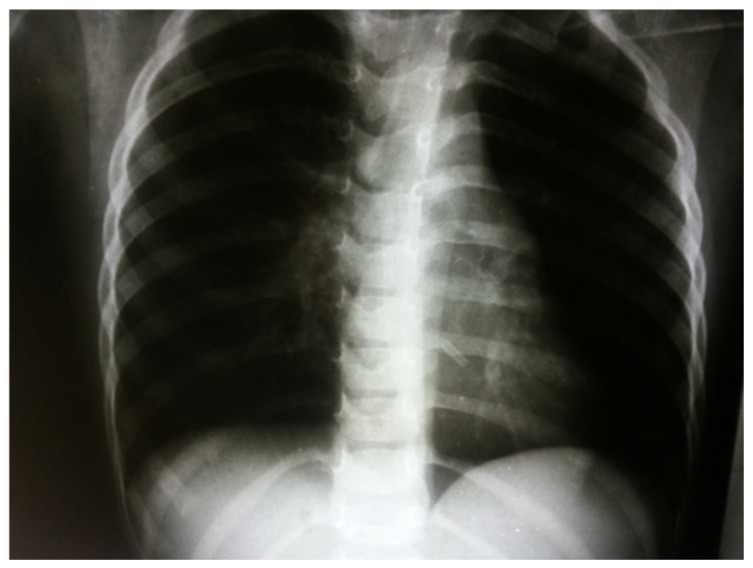
Pulmonary emphysema.

**Figure 2:**
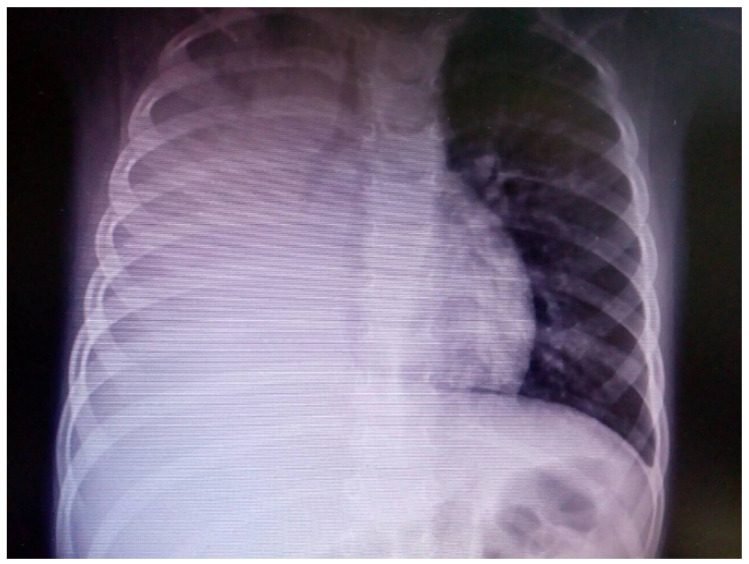
Pulmonary atelectasis.

**Figure 3:**
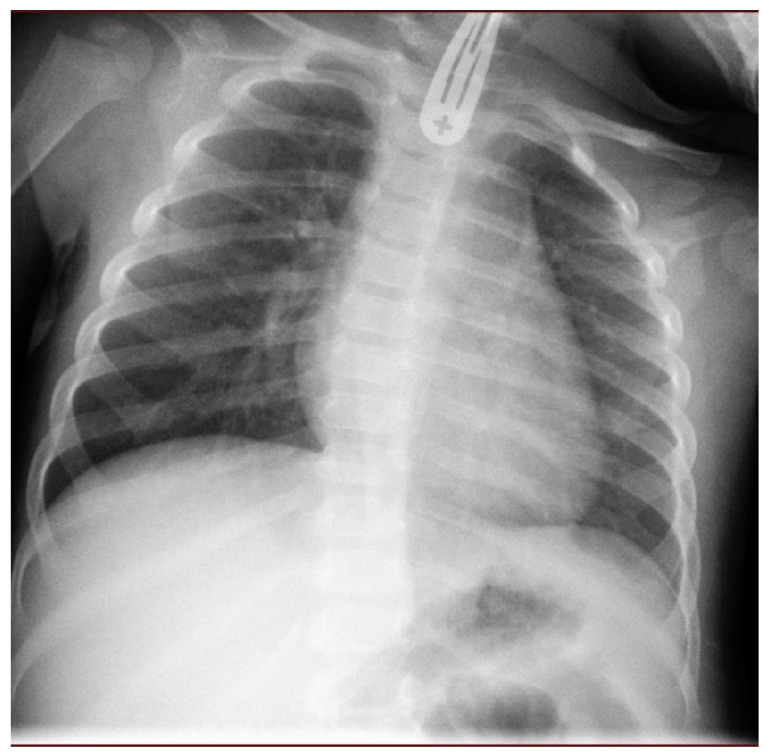
X-ray-positive foreign body.

Radiography is the main tool used to identify the presence of a foreign body in the respiratory tract, although most foreign bodies found there are X-ray-negative [1,6]. The ear, nose, and throat (ENT) surgeon must also consider that chest radiographs can be normal if they are obtained immediately after the aspiration of the foreign body because emphysema (Figure 1) or atelectasis (Figure 2) requires time to develop. However, the X-ray method is immediately conclusive if the item is X-ray-positive (Figure 3). Computer tomography (CT) imaging is rarely used for the diagnosis of acute foreign bodies in the respiratory tract. CT is usually indicated for the diagnosis of recurrent pneumonia, where foreign bodies are an incidental finding.

A possible aspirated foreign body must be rapidly diagnosed. The process is often performed under progressively urgent conditions, which may hinder the procedure. Fortunately, a bronchoscopy itself is the best diagnostic procedure for these situations. However, bronchoscopy is invasive, complex, may lead to complications, and requires highly specialized equipment and personnel, and that is why bronchoscopy is not the only diagnostic and therapeutic procedure used in such situations.

Flexible bronchoscopy has been increasingly applied, with the advances in medicine, for the diagnosis of various pulmonary diseases in children, including the detection of foreign bodies in the lower respiratory tract. In the 1990s, Wood et al. performed flexible bronchoscopy in children with a suspected foreign body in the trachea and bronchi but without clear evidence of foreign body aspiration.

Aspirated foreign bodies are extracted with the help of rigid and flexible bronchoscopy, the former of which is the main choice in Bulgaria. There was a rule that mandated that the child with a suspected foreign body in the respiratory tract attends the nearest hospital, and the bronchoscopy team travels to them, because of the well-developed hospital infrastructure covering the entire country. The idea was good because a foreign body in the airways can change in position while a child is transported, and the partial obstruction of the airways can progress to a total obstruction, leading to a fatal outcome.

This rule is no longer suitable today because personnel and equipment are lacking. The children are transported via specialized transportation to the hospital with a bronchoscopy team, where the extraction is performed as quickly as possible under general anesthesia.

A pediatrician usually examines the child again on the day after the foreign body is extracted. A control X-ray is performed in some cases. Children are usually discharged from the hospital 48 h after the extraction in the absence of a superimposed lung infection or complication caused by the procedure. The hospital stay is prolonged if inflammatory processes are detected; in the most severe cases, patients are admitted to the children’s pulmonology wards for further treatment. The child may be referred to an intensive care unit in isolated cases, mainly in the presence of spasm caused by the foreign body and the bronchoscopic intervention.

## 2. Materials and Methods

**Scheme 1:**
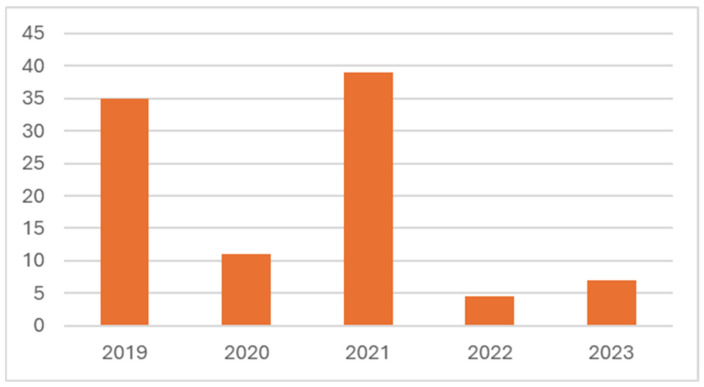
Five-year distribution of patients that underwent bronchoscopy.

A trained and well-equipped bronchoscopic unit covering a large part of Bulgaria has been operating for many years in the ENT clinic of the UMHAT St. George Plovdiv, in close relationship with the pediatric department. For a 5-year period, 154 children with a working diagnosis of a suspected foreign body in the respiratory tract were admitted and underwent bronchoscopy (Scheme 1).

**Scheme 2:**
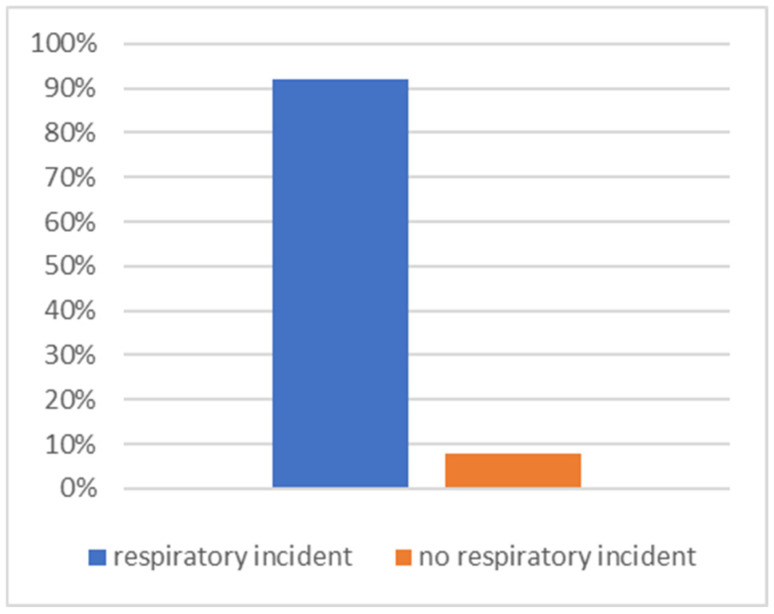
Presence/absence of anamnestic data on foreign body aspiration.

According to the anamnestic data, 92% of the patients had an incident leading to acute respiratory distress, and 8% had no definite data on such an event (Scheme 2). Recurrent pneumonia with the same localization raised suspicion of a chronic X-ray-negative foreign body in the relevant lung area in five cases, which was the reason for the bronchoscopy.

The following algorithm was applied for all patients:

1. Taking a detailed history from the adults accompanying the child and by the medical team, if brought by ambulance or sent from another medical facility;
2. Obtaining permission from the child’s caregiver(s) to perform emergency bronchoscopy;
3. Quick admission, processing, and basic tests, such as blood tests and chest X-ray;
4. Urgent consultation with a pediatrician (on site) and an anesthesiologist; and
5. Assembling a team and performing the bronchoscopy as soon as possible after admission.

Deviations from this algorithm occurred in isolated cases, usually involving performing bronchoscopy without waiting for the tests and/or pediatric consultation due to worsening dyspnea.

During this 5-year period, a number of children with a history of an acute respiratory incident (probable aspiration of a foreign body) whose parents refused hospitalization and left the hospital also attended the emergency ENT office. They were excluded from the statistics.

**Figure 4:**
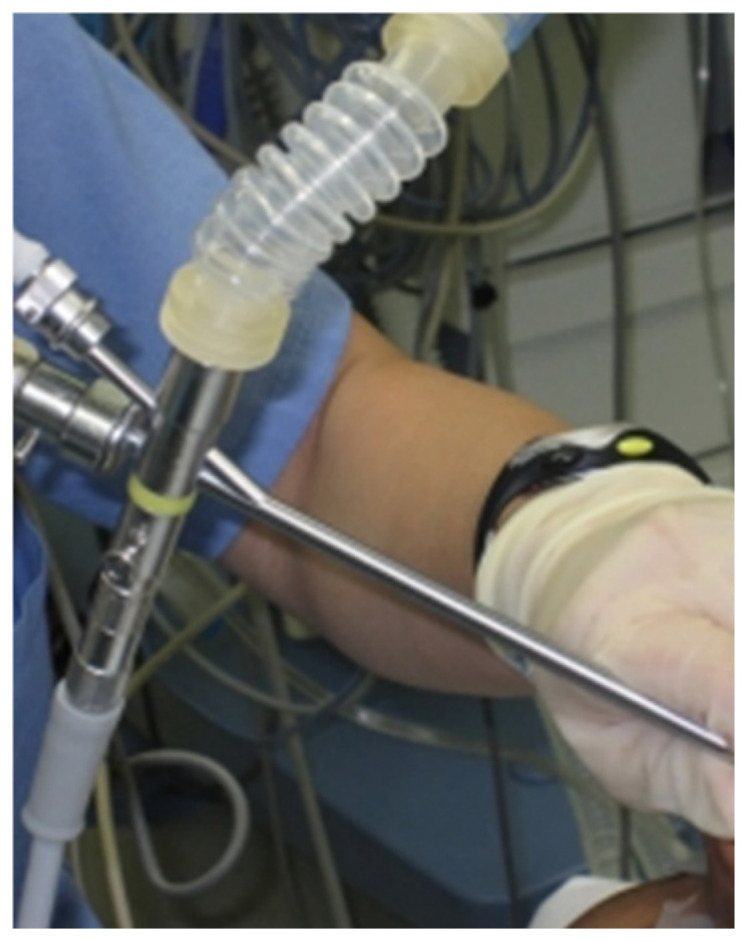
Rigid Storz bronchoscope.

**Figure 5:**
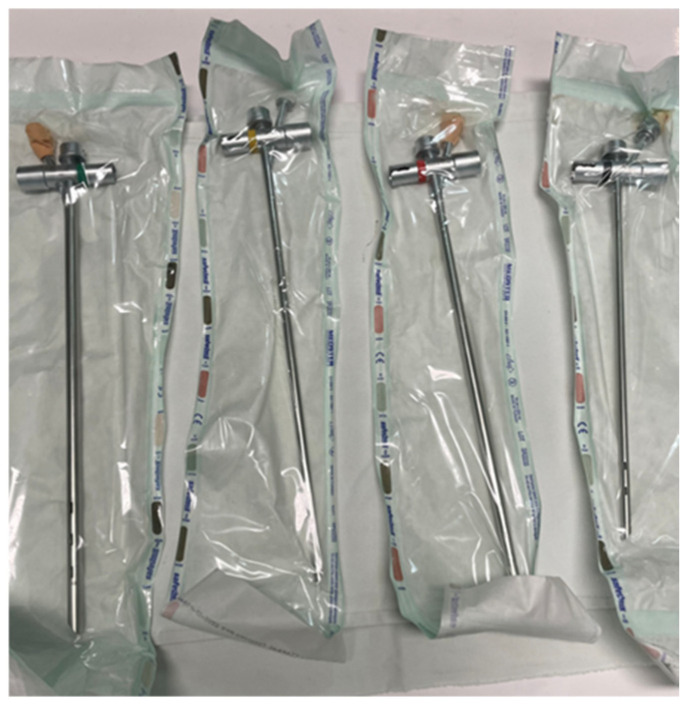
Rigid Storz bronchoscope.

**Figure 6:**
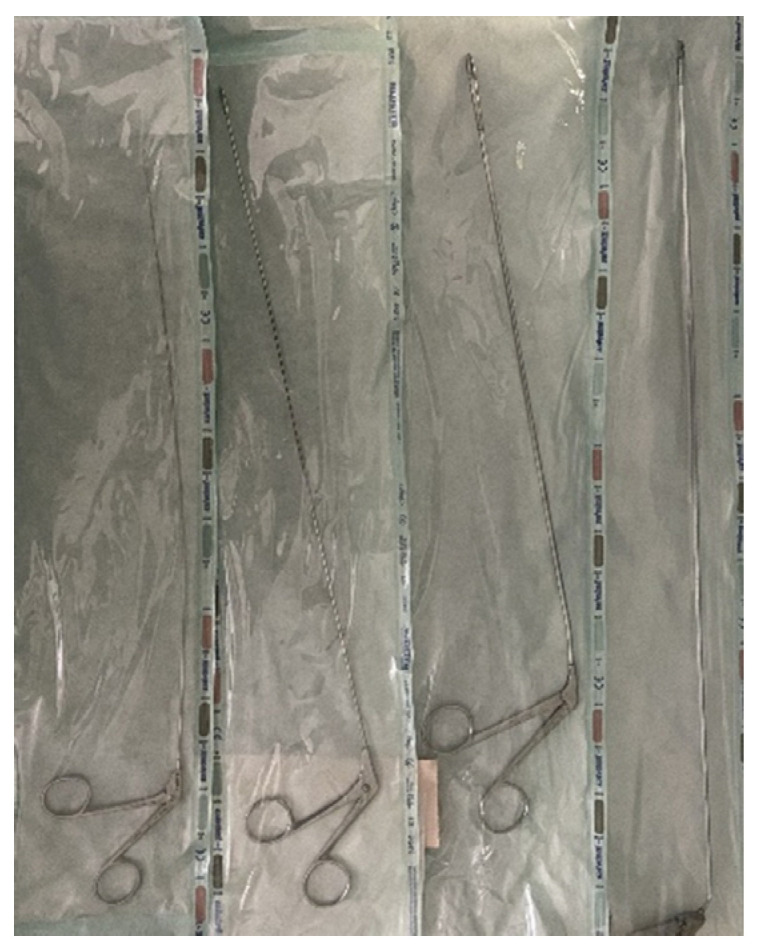
Extraction forceps.

The bronchoscopic examination and extraction were performed using a rigid (STORZ Germany model) bronchoscope (Figure 4 and Figure 5) and a set of bronchoscopic extraction forceps (Figure 6).

**Scheme 3:**
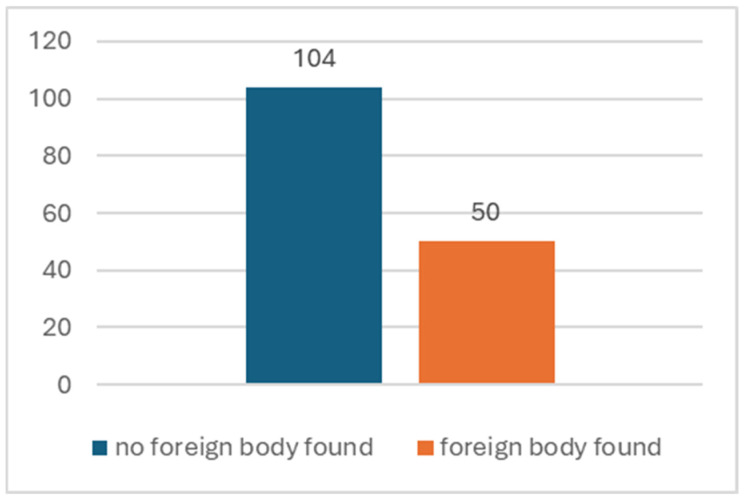
Distribution of patients according to presence/absence of a foreign body upon bronchoscopy.

A foreign body in the respiratory tract was found and extracted in 50 young patients; no foreign body was found in 104 of the cases, according to the statistical bronchoscopy data (Scheme 3).

**Scheme 4:**
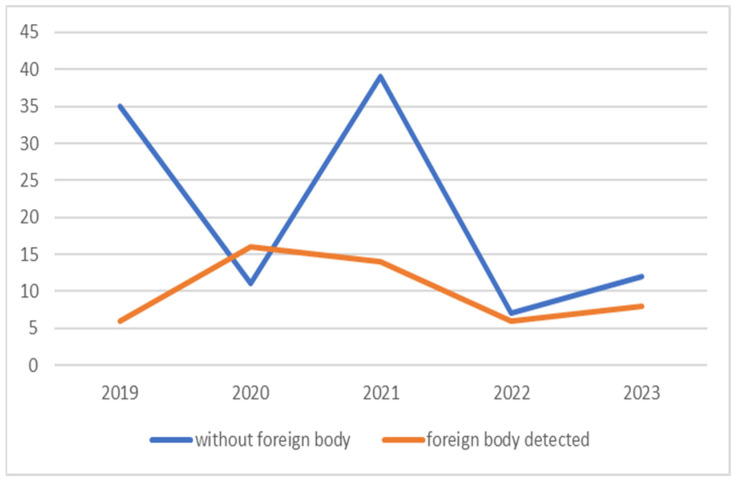
Presence/absence of a foreign body in children undergoing bronchoscopy over time.

The distribution of the presence/absence of a foreign body over time is presented in Scheme 4.

**Figure 7:**
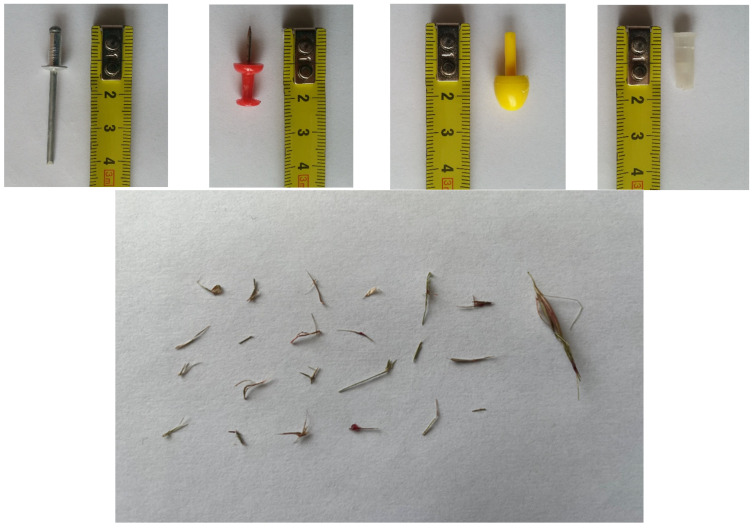
Extracted foreign bodies.

The extracted foreign bodies were of all material types and shapes, as shown in Figure 7.

No fatality due to foreign body aspiration in children was recorded in the study period (2019–2023). The foreign body was successfully extracted in all 50 cases. In twenty cases, the bronchoscopy was repeated due to the suspicion that a part of the foreign body remained after the initial examination and extraction (biological foreign bodies). Of these, a remnant was found and extracted in three. The patients were mostly discharged within 48 h of the bronchoscopic examination after a repeat consultation with a pediatrician and, in several cases, after a repeat chest X-ray.

A total of 21 bronchoscopied children required an extended hospital stay due to mild spasms or inflammatory changes in the lung. Six children were transferred to the pediatric department for further treatment due to severe inflammatory changes in the lung parenchyma. Four patients spent 24 h after the bronchoscopy in a pediatric intensive care unit and were transferred back to the ENT or pediatric department for further treatment (Table 1).

## 3. Discussion

Although the human body is equipped with a number of protective mechanisms to maintain free and clear airways, foreign bodies often fall into and remain in the airway, especially in childhood [7].

An urgent pediatric bronchoscopy performed due to the suspicion of a foreign body in the lower respiratory tract is a stressful procedure for the body of a child, the caregivers, and the bronchoscopist. Our statistics showed a slight decrease in the number of these cases, but the number of cases fluctuated over time. The data also indicate the overdiagnosis of foreign body aspiration in childhood due to the correct medical rationale that an “extra” bronchoscopy is better than an undiagnosed foreign body in the lower respiratory tract in children. However, bronchoscopy is a risky procedure, being performed under general anesthesia, that should be seriously considered prior to recommendation.

Rigid bronchoscopy is the golden standard method of choice for detecting and extracting foreign bodies from the airways. However, the literature review and our experience show that the rate of negative rigid bronchoscopic findings is variable, ranging from 7 to 46% [8,9].

The authors have recently used a combination of flexible and rigid bronchoscopy, particularly in cases of suspected foreign body aspiration. Flexible bronchoscopy is increasingly being used as the initial diagnostic procedure in children with an uncertain history of choking, in the absence of physical and radiological lung changes, and in cases with chronic complaints requiring the exclusion of a foreign body in the lower airways. Rigid bronchoscopy is directly performed in patients with clear evidence of foreign body aspiration based on physical and radiographic findings [8,9,10,11].

Up to 40% of patients may have no physical findings [5], and between 7 and 21% of children with foreign bodies have normal X-rays [11]. A history of “choking” is an important indicator of respiratory distress. This requires bronchoscopy to detect a foreign body in the airways, regardless of the presence or absence of physical and radiographic changes [11].

In our study, 92% of patients reported an aspiration incident, but rigid bronchoscopy identified foreign bodies in only 33% of them (67% of the cases were foreign-body-negative). The use of flexible bronchoscopy as an initial diagnostic method in uncertain cases will reduce the negative results in rigid bronchoscopy [8,10,11].

## 4. Conclusions

Foreign bodies entering the respiratory tract of children remains a common problem. No significant reduction has occurred in foreign body aspiration incidents in children worldwide, as also supported by our data. Foreign bodies in the respiratory tract still result in significant mortality among the young population despite the advances in diagnostic and extraction methods [1,12].

Immediate and adequate actions are required in cases of foreign body aspiration. Equipment and trained personnel must be available for these tasks, and the procedures established for such situations must be followed.

The main principle that one “extra” bronchoscopy is better than a missed foreign body should be followed in every suspected case because approximately 40% of the patients are asymptomatic and present no alterations upon physical examination.

The use of flexible bronchoscopy for initial diagnosis is a matter of choice; rigid bronchoscopy should be preferred for foreign body extraction because of its lower risk of complications [13].

Large-scale educational programs are required for young parents (especially from minority groups) to reduce the number of aspirated foreign bodies in children [14]. Additionally, the training of pediatricians and general practitioners could shorten the period from incident to diagnosis, which would reduce the interval from aspiration to extraction of the foreign body.

## Figures and Tables

**Table 1:** Distribution of bronchoscopy in children.

Foreign-body-negative	104 cases
Foreign body extracted	50 cases
Second bronchoscopy	20 cases (3 cases foreign-body-positive and 17 foreign-body-negative)
X-ray exam after bronchoscopy	5 cases
Hospital stay less than 24 after bronchoscopy	123
Hospital stay more than 24 h after bronchoscopy	21
Admission to pediatric department for further treatment	6
Pediatric intensive care unit admission after bronchoscopy	4

## Data Availability

The original contributions presented in the study are included in the article, further inquiries can be directed to the corresponding author.
